# Identification of Upper-Limb Movements Based on Muscle Shape Change Signals for Human-Robot Interaction

**DOI:** 10.1155/2020/5694265

**Published:** 2020-04-14

**Authors:** Pingao Huang, Hui Wang, Yuan Wang, Zhiyuan Liu, Oluwarotimi Williams Samuel, Mei Yu, Xiangxin Li, Shixiong Chen, Guanglin Li

**Affiliations:** ^1^CAS Key Laboratory of Human-Machine Intelligence-Synergy Systems, Shenzhen Institutes of Advanced Technology (SIAT), Chinese Academy of Sciences (CAS), Shenzhen 518055, China; ^2^Guangdong-Hong Kong-Macao Joint Laboratory of Human-Machine Intelligence-Synergy Systems, Shenzhen 518055, China; ^3^University of Chinese Academy of Sciences, Beijing 100049, China

## Abstract

Towards providing efficient human-robot interaction, surface electromyogram (EMG) signals have been widely adopted for the identification of different limb movement intentions. Since the available EMG signal sensors are highly susceptible to external interferences such as electromagnetic artifacts and muscle fatigues, the quality of EMG recordings would be mostly corrupted, which may decay the performance of EMG-based control systems. Given the fact that the muscle shape changes (MSC) would be different when doing various limb movements, the MSC signal would be nonsensitive to electromagnetic artifacts and muscle fatigues and maybe promising for movement intention recognition. In this study, a novel nanogold flexible and stretchable sensor was developed for the acquisition of MSC signals utilized for decoding multiple classes of limb movement intents. More precisely, four sensors were used to measure the MSC signals from the right forearm of each subject when they performed seven classes of movements. Also, six different features were extracted from the measured MSC signals, and a linear discriminant analysis- (LDA-) based classifier was built for movement classification tasks. The experimental results showed that using MSC signals could achieve an average recognition rate of about 96.06 ± 1.84% by properly placing the four flexible and stretchable sensors on the forearm. Additionally, when the MSC sampling rate was greater than 100 Hz and the analysis window length was greater than 20 ms, the movement recognition accuracy would be only slightly increased. These pilot results suggest that the MSC-based method should be feasible in movement identifications for human-robot interaction, and at the same time, they provide a systematic reference for the use of the flexible and stretchable sensors in human-robot interaction systems.

## 1. Introduction

In recent years, wearable devices [1, 2], such as exoskeletons and prostheses [3, 4], have shown a substantial promise in the fields of healthcare and rehabilitation that focus on restoring upper or lower extremity motor functions. More so, advances in technology have led to the development of wearable devices in the form of smart electronics that could continuously monitor different physiological parameters associated with the health status in humans [5, 6]. Although such wearable systems, especially the exoskeletons and prostheses, have been well developed for decades with remarkable advancements, their commercial and clinical success are still marginal. One of the reasons for this issue should be that the motion intention recognition mechanism employed by the devices is inconsistently accurate, thus leading to poor control output when utilized in a real-life scenario. Meanwhile, accurate motion intention recognition mechanism constitutes an essential part of the devices. Surface electromyogram (sEMG) and electroencephalogram (EEG) have been commonly considered as potential sources of biosignals from which information for decoding human limb movement intents can be seamlessly obtained, due to their noninvasiveness and ease of acquisition. Although these physiological signals have been widely utilized, they are relatively weak and susceptible to various kind of interferences. For instance, power line noise and motion artifacts would inevitably degrade the motion intention recognition accuracy of wearable systems that utilize sEMG or EEG signals as their sources of control. In an attempt to address this issue, researchers have sort alternative means from which motion intentions could be decoded which includes ultrasound [7], pressure [8], capacitance [9], muscle circumference [10], and muscle activation [11, 12]. However, some of the systems are relatively large in size and integrate sensors that lack flexibility and stretchability characteristics, which are the core requirements for developing smart miniaturized intelligent devices that could be easily adopted in practical applications. Therefore, there is a need to conduct further research in this direction that would lead to the development of a new sensing material for motion intention recognition with the capability to resolve the limitations of the existing sensing techniques in the context of wearable systems.

Recently, the use of flexible and stretchable sensing materials had attracted much attention in the bioelectrical signal recording and health monitoring domains [13, 14]. In this regard, various flexible and stretchable sensors have been developed [15], including strain, pressure [16–21], and tactile [22, 23] sensors. Interestingly, these sensors have been used for human motion monitoring [24–29], human-machine interfaces in the context of rehabilitation, and health monitoring [30–33]. Compared to the traditional sensors, some of these sensors are not only flexible and stretchable but also equipped with additional new features including self-power, self-cleaning, self-healing, and transparency, making them more convenient and feasible to adopt in the modern-day wearable systems. For instance, Song and Yang developed a self-power sensor with the capability to monitor human body movements while sleeping [34]. A self-healing strain sensor was developed by Cai et al. to detect the different joint movements in humans [35]. Trung et al. proposed a transparent hybrid sensor that could detect the temperature and strain associated with the human body [36]. In another study, Muth et al. developed a strain sensor that was mounted on a glove to detect the movements of human fingers [37], while Meyer et al. proposed the use of a textile pressure sensor for the detection of muscle activities in human [38]. It should be noted that the above work mainly focused on examining the electrical and physical properties of the sensors without systematic investigation and detailed experimental study of the sensors particularly in the context of human motion intention recognition, which constitutes a research gap.

To fill this research gap which may facilitate practical applications, this study firstly developed a new sensor based on nanogold flexible material to detect muscle shape change (MSC) information from which limb movement intentions could be adequately decoded. Secondly, a portable wireless acquisition system was built for the recording of the MSC signals picked up by the nanogold flexible and stretchable sensors. Thirdly, the performance of the newly developed MSC-based sensor for motion intention recognition was extensively validated following a systematic study using datasets obtained from nine able-bodied subjects that observed seven classes of targeted upper-limb movements. Fourthly, we investigated the effects of sensor dimension, placement location, sampling rate, feature extraction method, and analysis window length, on the motion intention recognition accuracy (this is the main index for evaluating the performance of the system) of the proposed MSC-based sensor. Lastly, the stretchability and flexibility of the proposed motion intention recognition MSC-based sensor were also examined to determine the possibility of adopting it in real-life applications. In summary, we believe that this study would provide a symmetric guide on the selection of optimal core parameters (such as feature set, locations, sizes, sampling rates, and window lengths for data processing) required in the practical application of stretchable and flexible sensor in the context of motion intension recognition for human-robot interaction.

The rest of this paper is organized as follows.  Section 2 describes the fabrication process of nanogold flexible and stretchable sensor and the portable wireless acquisition system and gives the systematic experimental protocols utilized in validating the sensor's characteristics.  Section 3 presents the experimental results.  Section 4 discusses the results. Finally, Section 5 presents the conclusion and future work.

## 2. Materials and Methods

### 2.1. Material

**Figure 1:**
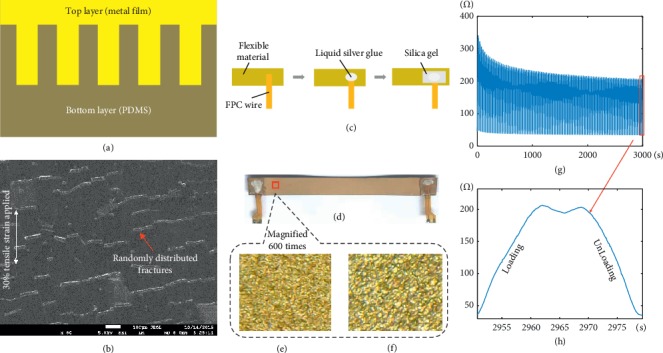
Fabrication of the sensor. (a) The structure of the material. (b) The microcracks of the gold film. (c) The process of fabrication. (d) The sensor. (e) The free state. (f) The stretched state. (g) One hundred circles of testing. (h) One circle of loading and unloading.

Gold is a well-known material with characteristics such as good conductivity, ductility, and biocompatibility, while polymers are soft and stretchable with good biocompatibility. With the aid of the state-of-the-art nano and microprocessing technology, we developed a soft and stretchable conductor using gold and polymer materials, which is conformal and biocompatible to detect biomechanical signal induced by the shape change of muscles. A detailed description of the fabrication process of the material is described in [39]. Meanwhile, the structure of the material used to fabricate the soft-stretchable sensor is presented in Figure 1(a). As shown in this figure, the top layer is made of the nanogold film while the bottom layer is a substrate known as polydimethylsiloxane (PDMS). Randomly distributed microcracks were observed inside the thin metal film on top of the polymer, as shown in Figure 1(b). When the film is subjected to tensile strain, the conductive path is still built up due to the randomly distributed microcracks. In addition, the conductivity of the material changes regularly with the opening and closing of the microcracks during the stretch/release process.

Since the conducting material is soft and stretchable and conformal with the texture of the human skin surface, the MSC can effectively induce the corresponding mechanical strain in the conductor. Additionally, the stretchable conductor maintains the conductivity regularly during the mechanical tensile strain, and by examining the conductivity during the strain, the corresponding changes with respect to the muscles' shape can be detected.

### 2.2. Fabrication of the Sensors

The sensors utilized for acquiring the MSC signals in this study were fabricated as follows. Firstly, the nanogold material was cut into strips with each strip having a length and width of approximately 8 cm and 8 mm, respectively. Thereafter, flexible printed circuit board (FPC) wires were attached to the strips through a liquid silver gel at the two terminals, and then the strips were placed in an open space for about 10 minutes so that the wires could get glued to the strips properly. This procedure is represented in Figure 1(c). After the silver glue dried up, a silica gel was applied over the silver gel to enhance its adhesiveness which would protect the encapsulated regions of the strips. Finally, the fabricated sensors were placed in a curing oven at 60°C for six hours. A representative of the resulting sensor is shown in Figures 1(d)–1(f), characterized by a stretched surface area which is 600 times the original surface area of the strip. The stability of the fabricated sensors was examined by pulling the strips 100 cycles on a tensile machine (AG-X plus 100N, Shimadzu, Japan) and a multimeter (Keithley 2000, Tektronix, USA) to observe the stretchability against its resistance. The pulling process is described as follows: (a) the sensors were pulled at a constant speed of 1 mm/s along the direction of the sensors; (b) When the sensors were stretched at the speed of 1 mm/s to the predetermined elongation (20% of their lengths), the tensile machine held the state for 5 seconds. Ater that, the tensile machine relaxed at the speed of 1 mm/s until the sensors recovered to their original lengths. As shown in Figures 1 (g)and 1(h), when the number of stretching cycles increases from 1 to 100, a corresponding decrease from an initial 340 ohms to a stable 210 ohms in the resistance of the sensor is observed. This stretching process makes the sensors more consistently stable for practical applications.

### 2.3. MSC Signal Acquisition System

**Figure 2:**
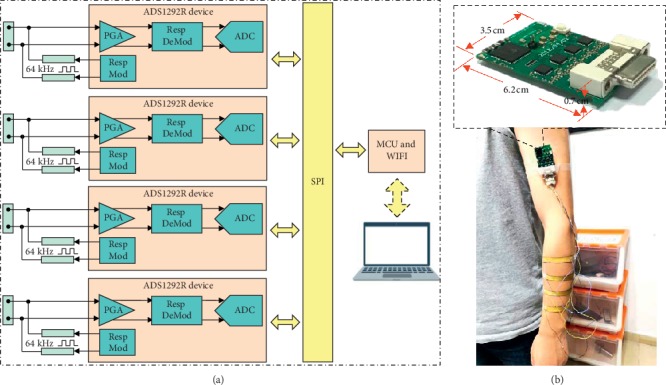
The data acquisition system. (a) The schematic diagram of the acquisition system. (b) The practical system.

A 4-channel acquisition system (length: 6.2 cm, width: 3.5 cm, height: 0.7 cm, and weight: 19 g) was developed to obtain the alteration in resistance of the sensors caused by the muscle shape change. As shown in Figure 2, the acquisition system was made up of three parts, namely, the analog front-end module, an MCU (WIFI transceiver included) module, and a computer. The ADS1292R (Texas Instruments, Texas, USA) is the analog front-end chip used to acquire bioelectrical signals, such as EMG signals. This analog front-end chip is also used for respiration resistance measurement. The respiration modulating module generates a 64 kHz square wave that is applied to the sensor, thus inducing a current that flows through the sensor. Thereafter, a voltage is produced by the current in the sensor and then amplified and demodulated by a respiration demodulating module. The demodulated signal is then digitalized by using a 24 bits sigma-delta ADC, and finally, the data are sent to the computer through WIFI module.

### 2.4. Setup of Experiments

To investigate the performance of the newly fabricated sensors, two different experimental sessions were designed for the collection of MSC signals associated with multiple classes of upper-limb movements in an offline mode. Information about the participants and the data acquisition procedure is provided as follows.

#### 2.4.1. Participants' Information

In this study, a total of nine able-bodied subjects including six males and three females (aged from 24 to 30, with an average of 26.3) were recruited. The protocol of this study was approved by the Institutional Review Board of Shenzhen Institutes of Advanced Technology, Chinese Academy of Sciences. All subjects gave written informed consent and provided permission for the publication of their photographs for scientific and educational purposes.

#### 2.4.2. Setup of the Movements

**Figure 3:**
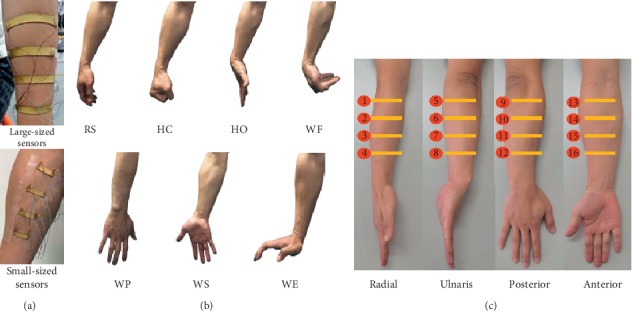
The protocol of the experiments. (a) Placements of two types of sensors on the forearm. (b) Seven targeted movements. (c) Sixteen locations.

The MSC signals were acquired at a sampling frequency of 1000 Hz using the four-channel data acquisition system described above. More precisely, two different kinds of stretchable-flexible sensors, large-sized sensors (length: 8 cm and width: 0.8cm) and small-sized sensors (length: 3 cm and width: 5.0 mm), were designed for the MSC data collection with an attempt to see if the sensor size would affect the MSC recordings, as shown in Figure 3(a). During data collection sessions, each participant was instructed to perform seven classes of targeted upper-limb movements that were hand close (HC), hand open (HO), wrist pronation (WP), wrist supination (WS), wrist extension (WE), wrist flexion (WF), and one inactive limb movement known as the rest state (RS) as shown in Figure 3(b). Note that these classes of the upper-limb movement tasks have been considered in a number of previous related studies [40–42]. Prior to the data collection sessions, the subjects were properly instructed about the experimental procedure to guarantee high-quality recordings. Furthermore, each subject was allowed to perform several preexperimental trials to get themselves familiar with the experimental protocol. Following these procedures, the subjects performed each movement based on a video prompt for 5 seconds, and each movement class was followed by a rest session of five seconds before observing the next active movement class. In training, the order of active movements is as follows: HC, HO, WP, WS, WE, and WF, and each subject was asked to repeat the process three times.

#### 2.4.3. Locations of the Sensors

In order to examine the optimal location for the MSC sensor placement, 16 locations along the vertical plane were selected. This is because if placed along the longitudinal direction of the arm, the sensors would be folded and therefore capture lesser information since they will not be making absolute contact with forearm muscles. As shown in Figure 3(c), each column's sensors were equally distributed between the chelidon and the end (near the hand) of the brachioradialis muscle. For the large-sized sensors, different placement locations, namely, the radial side (column 1, sensors 1 to 4, named region 1), ulnaris side (column 2, sensors 5 to 8, named region 2), posterior side (column 3, sensors 9 to 12, named region 3), and the anterior side (column 4, sensors 13 to 16, named region 4) of the forearm, were designed. For the small-sized sensors, four additional sensor placement strategies were used, but this time in a rowwise manner. The small-sized sensor placements are described as row 1 (sensors 1, 5, 9, and 13, named region 5), row 2 (sensors 2, 6, 10, and 14, named region 6), row 3 (sensors 3, 7, 11, and 15, named region 7), and row 4 (sensors 4, 8, 12, and 16, named region 8). It should be noted that the four sensors in a row assume a ring shape around the forearm as shown in Figure 3(c). Using the self-made four-channel acquisition system, the MSC signals could be measured in one row or one column on the forearm. For the large-sized sensors, the data from one experiment could be collected in four times, and the data from one experiment could be collected in 8 times for the small-sized sensors. During the experiment, each subject was asked to take a rest for two minutes between two acquisition sessions. Before being used, each sensor was prestretched and then adhered to the skin with medical adhesive tapes to ensure that they were firmly fixed to the skin during the experimental trials.

#### 2.4.4. Data Preparation

After the MSC signals were acquired, a five-point moving average filter was applied to attenuate the inherent noise. Then, the filtered MSC data were downsampled from 1000 Hz to 500 Hz, 250 Hz, 100 Hz, 50 Hz, 40 Hz, and 20 Hz, respectively. Finally, to evaluate the effect of different window lengths on the accuracy, a series of windows of 20 ms, 50 ms, 100 ms, 200 ms, and 300 ms were used to segment the MSC data with the overlap length of half of their window lengths.

#### 2.4.5. Feature Selection and Classification

For each of the windowed MSC signals, six features (the mathematical expressions are shown in Table 1), mean value (MVAL), root mean square (RMS), simple square integral (SSI), third moment (TM3), logarithm detector (LOGD), and standard deviation (STD), were extracted, which were used in some previous studies [42, 43]. With the feature sets, the principal component analysis technique was applied to remove redundant information, and then a five-fold cross validation was utilized to partition the feature vector into a training set and a testing set. A linear discriminant analysis (LDA) classifier was built for each subject to predict the limb-movement intents [44, 45]. The major consideration to use the LDA classifier is its computational efficiency coupled with its wide usage for the human-machine interface. The detailed operational procedure of the LDA algorithm could be referred to as [46].

#### 2.4.6. Statistical Analysis

To examine whether each of the five factors (feature, sensor size, sampling rate, location, and window length) of the sensor has an impact on the accuracy of movement classification, the one-way ANOVA with a post hoc analysis LSD was conducted in terms of mean classification accuracy, using the SPSS Statistical Modeling software (SPSS 22.0 IBM Corp., Chicago, IL). To perform one-way ANOVA, when designing experiments and grouping data, only one of the five factors was changed at a time while several other factors retained their typical values unchanged. A level of *p* < 0.05 was selected as the threshold for statistical significance with the null hypothesis that the classification accuracies achieved by one factor's changing (such as the frequency changing, 1000 Hz, 500 Hz) among the five factors are not significantly different from each other.

## 3. Results

### 3.1. Waveforms of MSC Signals

**Figure 4:**
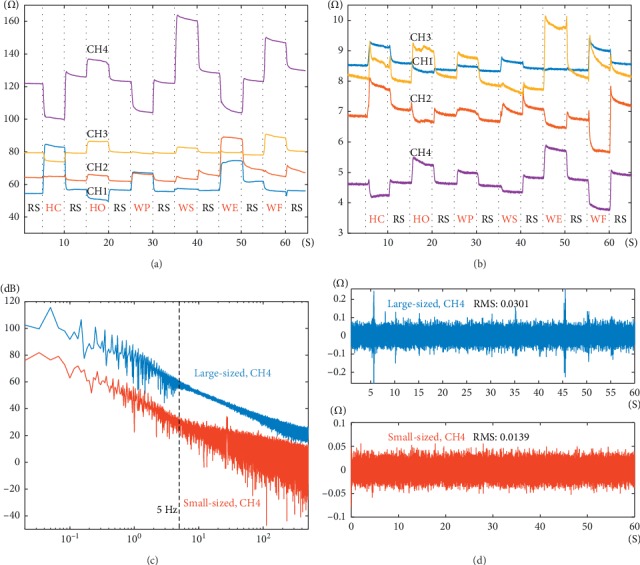
Typical waveforms of MSC signal recordings. (a) The 4-channel waveforms of the large-sized sensors. (b) The 4-channel waveforms of the small-sized sensors. (c) The signal spectrums of CH4 for both sensors. (d) The noises of CH4 for both sensors.

Figures 4(a) and 4(b) show two typical MSC recordings by the large-sized and small-sized sensors, respectively. The six classes of active movements and the inactive movement could be visually distinguished from the MSC recordings. The MSC signal values in different channels varied in a range of dozens of ohms during the movements and had a different baseline. The preapplied tension and the intrinsic resistance of the sensors were different from each other. At the rest state, MSC signal values seem different in different channels, varying with individual movement. Comparatively, the value of the large-sized sensors changed more than that of the small-sized sensors.

Figure 4(c) shows the spectrums of CH4 of both sensors. It could be known that the main components of the MSC signal are concentrated within 5 Hz. Therefore, the clean MSC signal *S*_MSC_ is obtained by using a low-pass filter (IIR, Butterworth, the cutoff frequency is 5 Hz), with very small noise (for the band is only 5 Hz). The noise *N*_*i*_ is obtained by a high-pass filter (IIR, Butterworth, the cutoff frequency is 5 Hz).  Figure 4(d) shows the noise of CH4 of both sensors, and the small-sized sensors have lower noise. Then, the SNR is calculated as equation (1), and the large-sized sensor and the small-sized sensor have SNRs of 49.10 ± 4.91 dB and 29.21 ± 1.97 dB, respectively. The SNR of the large-sized sensor is about 20 dB higher than that of the small-sized sensor.

(1) $$\begin{array}{c} \text{SNR}=10\;\log_{10}\frac{P_{S_{\text{MSC}}}}{{\text{P}}_{N_{i}}}. \end{array}$$

### 3.2. Effect of Features on the Classification Accuracy

**Figure 5:**
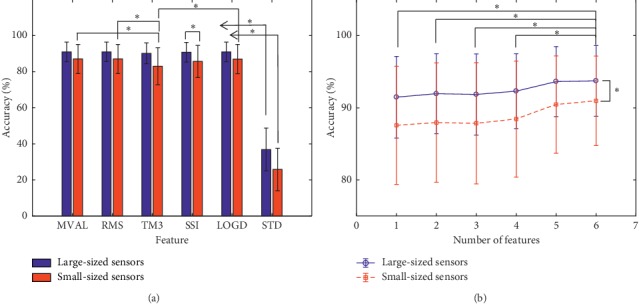
Effect of the features on movement classification accuracy. (a) Effect of different features on accuracy. (b) Effect of different numbers of features on accuracy.

Figure 5(a) shows the relationship between the average classification accuracy vs. the six features across all the sensor locations in nine able-bodied subjects with a sampling frequency of 1000 Hz and window lengths of 100 ms. For the two groups of sensors, the classification accuracies when using the STD feature were significantly different from the other features (*p* < 0.01). For the small-sized sensors, except for the STD feature, the TM3 reflected a different characteristic in comparison to the MVAL, RMS, and LOGD (*p* < 0.016) features, respectively. For the large-sized sensors, MVAL, RMS, TM3, SSI, and LOGD almost had the same accuracy and no significant difference (between all groups, *p* > 0.635). It can be seen from Figure 5(a) that the accuracy of the large-sized sensors was about 3% higher than that of the small-sized sensors. For all the six features, the average accuracies of the movement classifications were about 81.81 ± 21.29% when using the large-sized sensors and 75.95 ± 24.30% when using the small-sized sensors. ANOVA shows that the average classification accuracies were significantly different for the different sensor sizes (*p*=0.005).

Furthermore, using the MVAL feature as a basis, the other five features were added one-by-one in the sequence of RMS, STD, LOGD, TM3, and SSI, and then used to classify the movement intentions for each subject as shown in Figure 5(b). It can be seen from Figure 5(b) that the classification accuracies slightly increased as the number of features increases (from 1 to 6) for both the large-sized and small-sized sensor configurations. For the small-sized sensors, the first four features have no significant impact on accuracy (at, *p* > 0.50), while the addition of the fifth and sixth features led to a significant increase in accuracy (for five features, *p*=0.035; six features, *p*=0.013), from 87.57 ± 8.20% to 90.97 ± 6.19%. For the large-sized sensors, there is no significant difference in accuracy (*p* > 0.09) even when all the six features were concatenated and used for classifying the limb movement intent of the subjects, where only a slight increase can be observed in accuracy (from 91.47 ± 5.66% to 93.73 ± 4.90%). These results suggested that the large-sized sensors would achieve higher accuracy in comparison to the small-sized sensors (*p*=0.002).

### 3.3. Effects of Sensor Locations on the Classification Accuracy

**Figure 6:**
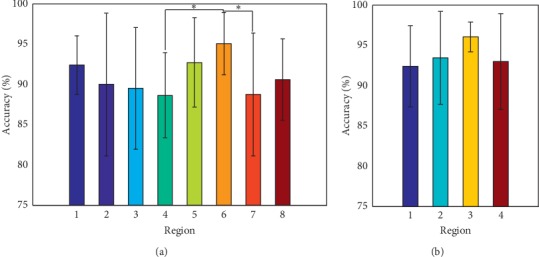
Accuracies vs. regions. (a) Accuracies of the small-sized sensors. (b) Accuracies of the large-sized sensors.

With an attempt to look for the optimal sensor placements on the forearm, the effects of different sensor locations on the forearm of the subjects were investigated. Eight different regions for the four small-sized sensors' placement and four different regions for the large-sized sensors' placement, as shown in Figure 3(b), were examined. Six features were used along with the sampling frequency and window length adopted in the previous section. The average classification accuracy over all the nine subjects was calculated with each sensor placement region and is presented in Figure 6. For the small-sized sensors, it can be seen from Figure 6(a) that the region 6 achieved the highest accuracy of 95.07 ± 3.87%, while the region 4 had the lowest accuracy of 88.64 ± 6.34%. Additionally, the ANOVA showed that there was significant difference between region 6 and region 4 (*p* = 0.042) and also between region 6 and region 7 (*p* = 0.046). Meanwhile, the total average accuracy (across all the subjects and all the regions) was about 90.97 ± 6.19%. For the large-sized sensors, the ANOVA indicated that no significant difference between all the four regions was observed (*p* > 0.15).  Figure 6(b) shows that region 3 had the highest average accuracy of 96.06 ± 1.84% among all the four regions, and the total average accuracy (across all the nine subjects and all the four regions) was about 93.73 ± 4.90%.

### 3.4. Effects of Sampling Rates and Window Lengths on the Classification Accuracy

**Figure 7:**
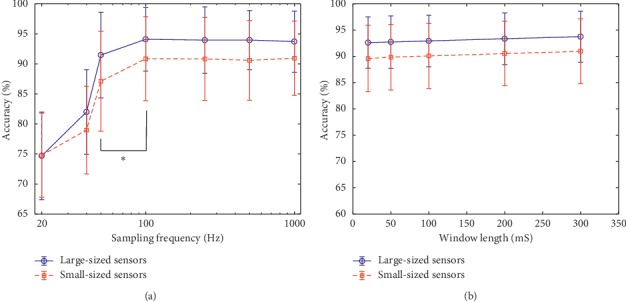
Effects of sampling rates and window lengths on movement classification accuracy. (a) Effect of sampling rates on accuracy (abscissa is logarithmic). (b) Effect of window lengths on accuracy.

To investigate the effect of different MSC signal sampling rates on the motion intention recognition accuracy, the acquired signal was downsampled from 1000 Hz to 500 Hz, 250 Hz, 100 Hz, 50Hz, 40 Hz, and 20 Hz, respectively. The identical window length of 300 ms was used for the different sampling rates and the six features were extracted from each analysis window. The overall classification accuracy over all the regions and all the subjects was calculated for each sampling rate, as shown in Figure 7(a). It can be observed from Figure 7(a) that using a sampling rate from 100 Hz to 1000 Hz, both the large-sized and small-sized sensors showed a steady accuracy. If the sampling frequency was lower than 50 Hz, there was an obvious and significant decrease in the accuracy (*p* < 0.001).

In addition, the effect of window length on motion intention recognition accuracy was examined by using five different window lengths (20 ms, 50 ms, 100 ms, 200 ms, and 300 ms), respectively. The six features were extracted from each analysis window with each window length for the motion intention recognition. The overall classification accuracies over all the regions and the nine subjects are shown in Figure 7(b). We can see from Figure 7(b) that the movement classification accuracies of both the large-sized and small-sized sensors only had a slight increment (less than 1.2%) with a corresponding increase in the window length.

### 3.5. Classification Accuracies of Different Movements

**Figure 8:**
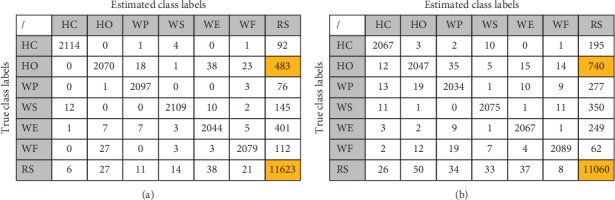
Confusion matrix. (a) Fusion matrix of large-sized sensors. (b) Fusion matrix of small-sized sensors.

Following the above-described procedures, to evaluate the classification performance of different movements, we calculated the confusion matrices of classification across all subjects for optimal sensor locations (Figure 8, in counts of testing samples). It can be known from the figure that (a) all active movements almost have the same true positives; (b) the movements HO and RS have the greatest interaction; (c) the samples of the classes are imbalanced (each active movement has 2133 samples, while the RS movement has 12933 samples; the ratio is about 1 : 6).

According to Sokolova and Lapalme, there are eight measures for multiclass classification, and the measures have some invariance properties (that is, they preserve their value under a change in the confusion matrix) [47]. For example, average accuracy is invariant to the exchange of positives and negatives of the confusion matrix, while recall is invariant to the change of true negative counts. This is beneficial for evaluating the performance of classification. Therefore, Precision (*P*_*i*_), Recall (*R*_*i*_), and F-score (*F*_*i*_) of each class (*β* = 1, Precision and Recall are considered equal) were calculated (shown in Table 2), and Macro-Precision (*P*_M_), Macro-Recall (*R*_M_), and Macro-F-score (*F*_M_) were also calculated (shown in Table 3). For both sensors, it can be seen from Table 2 that (a) the RS movement had the lowest Precision among all the features, but has the highest Recall; (b) the HO movement had the lowest Recall. (c) When considering both precision and recall, the HO movement had the worst performance. Additionally, all the three measures showed that the large-sized sensors could achieve relatively higher performance in comparison to the small-sized sensors (shown in Table 3).

## 4. Discussion

Adequate human motion intention recognition technique aids the realization of efficient human-robot interaction mechanisms required to provide intelligent control systems in the context of rehabilitation or service robots. Meanwhile, information extracted from a number of physiological signals such as EMG and EEG has been widely utilized for the decoding of human motion intention. However, such signals are often subjected to different interferences resulting from electromagnetic artifacts, touch resistance between skin and electrodes, and muscle fatigue among others. And these interferences have been well studied with reports revealing their negative effects towards degrading real-time performances of motion intention decoding. Alternatively, nonphysiological signals based on muscle geometric and/or morphology changes that can be measured by different techniques such as ultrasound [7], capacitance [9], muscle circumference [10], and muscle activation [11, 12] have been considered for motion intention recognition. In that regard, this study hypothesized that nonphysiological MSC signals should offer adequate information for limb movement intent decoding. In this study, we systematically examined the feasibility of utilizing the MSC signals acquired through the newly developed nanogold flexible and stretchable sensor for upper-limb movement intent decoding in the currently evolving human-robot interaction systems.

Firstly, we extensively examined the characteristics of the MCS signals when different features, namely, linear (MVAL), nonlinear (RMS, SSI, TM3, and LOG), and statistical (STD) features, were extracted for the limb movement decoding task under multiple criteria. The outcome of the investigation reveals that except for the STD feature that recorded extremely low accuracies (less than 50%), the other examined features achieved high accuracies with somewhat similar performance for the movement intent decoding task. Also, by concatenating the features extracted from the MSC signals in an incremental manner, it was found that using more features would only result in slight increase in accuracy (that is, about a 3.4% increase for the small-sized sensors and 2.26% increase for the large-sized sensors), indicating that it might be unnecessary to utilize multiple features when adopting the MSC signals in practical applications. Importantly, we found that regardless of the feature used to predict the movement intent of the subjects, they still exhibited similar waveforms except for their amplitudes that appeared to be different. Also, one or two features would be sufficient to achieve acceptable accuracy, which may minimize computational complexity.

**Figure 9:**
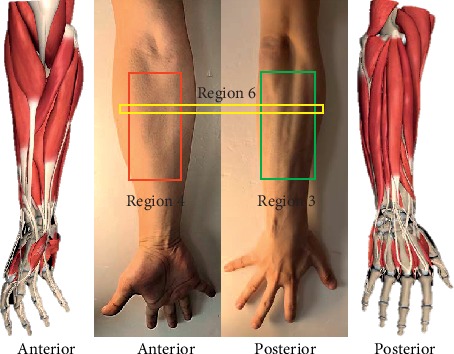
The muscles of the forearm.

Secondly, sixteen different forearm locations were mapped out to determine the most appropriate regions on the forearm for the sensor placement while considering two distinct sensor sizes (small-sized sensors and large-sized sensors). The experimental results showed that the sensors placed on the locations with more muscles led to higher accuracy in comparison to locations with fewer forearm muscles. Also, the small-sized sensors placed around region 6 achieved the highest accuracy as against the sensors placed around region 4 which recorded the lowest accuracy (Figure 6). This analysis is supported by Figure 9, in which the sensors in region 4 (sensors 13 to 16 of Figure 4) were placed in the center of the extensor digitorum, located at the anterior side of the forearm. Compared to the posterior side where there are seven superficial muscles, there are only four muscles in the anterior region. Thus, the sensors in region 4 may acquire less limb motion information in comparison to the other regions [48, 49]. In region 6 (row 2, sensors 2, 6, 10, and 14), the center of these four sensors was placed right on the bulges of all the muscles' bellies that had obvious shape changes when doing different movements, so these sensors may pick up the maximum shape change of the muscles and obtain relatively higher information than the sensors in the other locations. Thus, higher motion recognition accuracy could be achieved when utilizing MSC signals from region 6. For the large-sized sensors, the placement locations were similar to those of the small-sized sensors. Due to the relatively large surface area of the large-sized sensors, they cover more muscles and could capture more MSC information related to muscle activities. For example, sensor 7 of region 3 covers the brachioradialis, part of the extensor digitorum and part of the flexor carpi ulnaris. This is equivalent to the information obtained from the three small-sized sensors (sensors 11, 7, and 15). On the contrary, this makes the large-sized sensors less sensitive to local muscle information than their small-sized counterparts. This could explain the results presented in Figure 6(b).

Thirdly, two important parameters, the sampling rate and window length associated with processing the MSC signals, which accounts for the computational complexity of the entire motion intention recognition task, were also investigated. In the real-time applications (particularly for embedded microcontrollers) of the motion intention recognitions, a high sampling rate would normally lead to large computation time while a long window length often results in large delay. Thus, it is preferred to develop a system that has a lower sampling rate and adopts a shorter window length for the data processing task. As shown in Figure 7(a), sampling frequencies that are less than 100 Hz could be seen to affect the accuracy of the motion intention recognition classifier because the highest frequency of the MSC signal is between 20 and 50 Hz [50]. According to the Nyquist sampling theorem which states that the sampling frequency of a signal should be at least twice the signal's bandwidth [51], a sampling frequency of 100 Hz would be sufficient to preserve all the relevant information of the MSC signals. Therefore, utilizing sampling frequencies from 100 Hz or above could help maintain the motion intention recognition accuracy of the newly proposed MSC sensors. Meanwhile, Figure 7(b) shows that the window length has little effect on the motion intention recognition accuracy. In addition, it can be observed in Figure 4 that the MSC signal exhibits fewer changes while features extracted from longer windows yielded almost the same performance in terms of motion recognition accuracy compared to those extracted from shorter windows. In other words, varying the window length would only result in a slight increment in the motion intention recognition rate. Thus, the calculation amount can be further reduced by reducing the sampling rate, and the response time of the system can be improved by reducing the window length.

In summary, using the newly proposed MSC signal, one may realize a computationally efficient motion intention recognition system by considering a sampling frequency of about 100 Hz, a window length of about 50 ms, and one feature. The computational complexity of the intention recognition system is estimated as follows. It mainly includes three parts: (a) feature extracting. From Table 1, it can be known that the computational complexity is O(NM), where N is the total number of samples and M is the number of features (in this study, M < N); (b) training. According to [52], the computational complexity of LDA is O(NM^2^) when M < N; (c) classification. This step is the product of the coefficients and the data to be identified, so the computational complexity is O(NM). In total, the computational complexity of the system is about O(NM^2^). When one feature is used, the computational complexity is around O(N). In our system, N is a small number (about a few thousand when in training, and about a few hundred when in real-time application), so the system would be easily realized on a microcontroller and the system would be easily realized on a microcontroller.  Table 4 shows some results of the running time on our system (computer: Intel i5, Windows 7, MATLAB 2016; the large-sized sensors in region 1 of the nine subjects were tested with a window length of 100 ms). Sampling rate and number of features dramatically affect the time for feature extracting, and lower sampling rate and less features would decrease the computational complexity.

Despite the interesting results obtained in the current study, some issues were observed while analyzing the MSC signals. For instance, compared to the EMG signal, the MSC signal exhibited a relatively simpler waveform characteristic suggesting that it might contain relatively less information. Hence, it may be a challenge to recognize more classes of limb movements with high accuracy when using the proposed MSC signals. According to the work of Li et al. [53], most of the useful information from the EMG recordings for motion intention recognition is contained in the frequency range of 60 Hz to 250 Hz. Meanwhile, the proposed MSC signals have a relatively lower frequency of 50 Hz. Hence, a combination of these two kinds of signals (EMG and MSC signals) may provide complementary information in different frequency bands that would be potential for the development of accurately robust motion intention recognition system in real-life applications particularly when several targeted limb movements are to be decoded.

**Figure 10:**
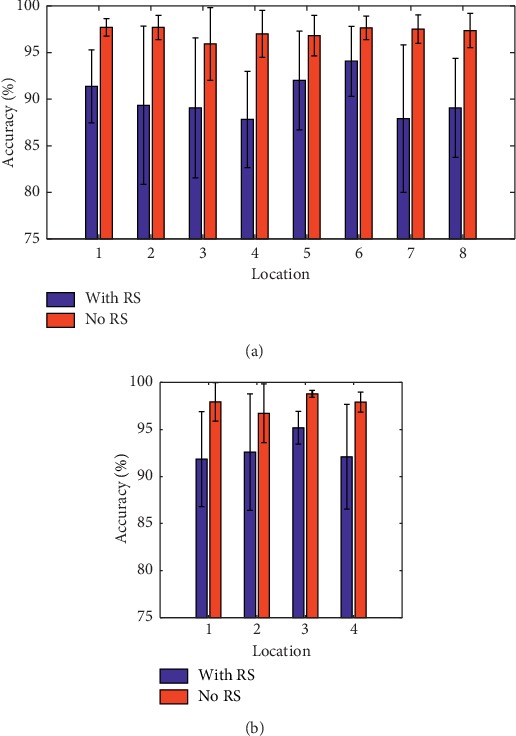
The RS was removed. (a) The accuracies of the small-sized sensors. (b) The accuracies of the large-sized sensors.

On the other hand, the MSC signals were characterized by creep [54], which causes crosstalk between the active and nonactive (RS) motion recordings, thus attenuating the motion recognition accuracy. This situation is particularly severe in cases where the RS and HO movements of the subjects are being predicted (Figure 8). Importantly, when data corresponding to the RS were excluded, higher motion intention recognition accuracies were achieved for both the small-sized and large-sized sensors across all locations (Figures 10(a) and 10(b))). Because the occurrence of the creep can be modeled using some methods [55], one possible solution would be to develop a creep-sensitive algorithm to reconstruct the MSC signal patterns according to a predefined model. Another possible way to resolve this issue would be to consider using EMG signal for detecting the RS states. This is because when the limb assumes a rest state, the amplitude of the EMG signal drops to around the baseline, and afterward, there is an obvious rise in the signal's amplitude when a targeted limb movement is elicited. Therefore, using EMG signal as a switch for the RS state, that is, only using EMG signal to identify the RS state, and using a combination of EMG and MSC signals to identify the active movement classes may lead to high and stable motion intention recognition in practical applications.

## 5. Conclusions

In summary, the proposed nanogold flexible and stretchable sensor was developed towards providing an alternative approach for motion intention recognition that may enhance the practical applications of pattern recognition systems. Based on the experimental results obtained in this study, accuracies of up to 95.07 ± 3.87% and 96.06 ± 1.84% were recorded for the small-sized and large-sized sensors, respectively, particularly when the sensors were placed at the optimal locations. Additionally, when using the proposed sensor for motion intention recognition, it often does not require a high sampling rate (just over 100 Hz) and a long window length (50 ms) for data processing. Interestingly, the newly proposed MSC sensor is not sensitive to feature set selection, indicating that simple feature methods could be applied to achieve an acceptable motion intention recognition accuracy in practical settings. Thus, this suggests that the proposed nanogold flexible and stretchable sensor would be feasible and effective in practical applications.

Despite the potential results obtained in the current study, there are still some shortcomings that need to be addressed in our future work. For instance, the issue of degradation in the performance of the proposed MSC sensors resulting from the creeping nature of the materials would hopefully be addressed through systematic investigation in our future work either through the development of intelligent signal processing algorithms or via a technique that would combine the MSC and EMG signals for motion intention recognition.

## Figures and Tables

**Table 1:** The mathematical expressions of the six features.

Serial number	Feature name	Abbreviation	Mathematical expression
1	Mean value	MVAL	MVAL=(1/*k*)∑_*n*=1_^*k*^*x*_*n*_
2	Root mean square	RMS	$\text{RMS}=\sqrt{\left(1/k\right)\sum_{n=1}^{k}{\left(x_{n}\right)}^{2}}$
3	Third moment	TM3	TM3=(1/*k*)∑_*n*=1_^*k*^(*x*_*n*_)^3^
4	Simple square integral	SSI	SSI=∑_*n*=1_^*k*^(*x*_*n*_)^2^
5	Logarithm	LOGD	LOGD=*e*^(1/*k*)∑_*n*=1_^*k*^(*x*_*n*_)^
6	Standard deviation	STD	$\text{RMS}=\sqrt{\left(1/k\right)\sum_{n=1}^{k}{\left(x_{n}-\text{MVAL}\right)}^{2}}$

**Table 2:** Precision, Recall, and F-score of each movement (unit: %).

	Measure	HC	HO	WP	WS	WE	WF	RS
Large-sized sensors	*P*_*i*_	99.10	97.05	98.32	98.86	95.81	97.45	**89.87**
	*R*_*i*_	95.59	**78.62**	96.27	92.58	82.83	93.45	**99.01**
	*F*_*i*_	97.31	**86.87**	97.28	95.62	88.85	95.41	94.22

Small-sized sensors	*P*_*i*_	96.89	95.96	95.34	97.3	96.89	97.93	**85.52**
	*R*_*i*_	90.73	**71.41**	86.11	84.69	88.63	95.22	**98.32**
	*F*_*i*_	93.71	**81.88**	90.49	90.56	92.58	96.56	91.47

**Table 3:** Macro-Precision, Macro-Recall, and Macro-F-score of both sensors (unit: %).

	*P*_M_	*R*_M_	*F*_M_
Large-sized sensors	96.64	91.19	93.65
Small-sized sensors	95.12	87.87	91.04

**Table 4:** Running time per subject (unit: ms).

(Hz)	Time for feature extracting		Time for training		Time for classification	
	One feature	Six features	One feature	Six features	One feature	Six features
1000	23.49 ± 6.57	165.8 ± 11.30	23.10 ± 3.88	20.79 ± 1.77	0.98 ± 0.45	0.86 ± 0.26
100	7.45 ± 3.95	33.79 ± 7.80	23.59 ± 3.85	22.15 ± 2.96	0.98 ± 0.37	0.69 ± 0.16

## Data Availability

The MSC data used to support the findings of this study are available from the corresponding author upon request.
